# Trends and outcomes for non-elective neurosurgical procedures in Central Europe during the COVID-19 pandemic

**DOI:** 10.1038/s41598-021-85526-6

**Published:** 2021-03-17

**Authors:** Lukas Grassner, Ondra Petr, Freda M. Warner, Michaela Dedeciusova, Andrea Maria Mathis, Daniel Pinggera, Sina Gsellmann, Laura C. Meiners, Sascha Freigang, Michael Mokry, Alexandra Resch, Thomas Kretschmer, Tobias Rossmann, Francisco Ruiz Navarro, Andreas Gruber, Mathias Spendel, Peter A. Winkler, Franz Marhold, Camillo Sherif, Jonathan P. Wais, Karl Rössler, Wolfgang Pfisterer, Manfred Mühlbauer, Felipe A. Trivik-Barrientos, Sebastian Rath, Richard Voldrich, Lukas Krska, Radim Lipina, Martin Kerekanic, Jiri Fiedler, Petr Kasik, Vladimir Priban, Michal Tichy, Petr Krupa, Tomas Cesak, Robert Kroupa, Andrej Callo, Pavel Haninec, Daniel Pohlodek, David Krahulik, Alena Sejkorova, Martin Sames, Josef Dvorak, Petr Suchomel, Robert Tomas, Jan Klener, Vilem Juran, Martin Smrcka, Petr Linzer, Miroslav Kaiser, Dusan Hrabovsky, Radim Jancalek, Vincens Kälin, Oliver Bozinov, Cedric Niggli, Carlo Serra, Ramona Guatta, Dominique E. Kuhlen, Stefan Wanderer, Serge Marbacher, Alexandre Lavé, Karl Schaller, Clarinde Esculier, Andreas Raabe, John L. K. Kramer, Claudius Thomé, David Netuka

**Affiliations:** 1 Department of Neurosurgery, Medical University Innsbruck, Anichstrasse 35, MZA 3rd floor, 6020 Innsbruck, Austria; 2 International Collaboration On Repair Discoveries (ICORD), University of British Columbia, Vancouver, BC Canada; 3 Department of Neurosurgery and Neuro-Oncology, 1St Medical Faculty, Charles University, Central Military Hospital, Prague, Czech Republic; 4 Department of Neurosurgery, Inselspital Bern, Bern University Hospital, Bern, Switzerland; 5 Department of Neurosurgery, Landeskrankenhaus Feldkirch, Feldkirch, Austria; 6 Department of Neurosurgery, Medical University Graz, Graz, Austria; 7 Department of Neurosurgery & Neurorestauration Klinikum Klagenfurt, Klagenfurt, Austria; 8 Department of Neurosurgery, Kepler University Hospital GmbH, Johannes Kepler University, Linz, Austria; 9 Department of Neurosurgery, Christian Doppler Clinic, Paracelsus Medical University, Salzburg, Austria; 10 Department of Neurosurgery, University Hospital of St, Karl Landsteiner University of Health Sciences, St. Poelten, Austria; 11 Department of Neurosurgery, Medical University of Vienna, Vienna, Austria; 12 Department of Neurosurgery, Klinik Donaustadt, Vienna, Austria; 13 Department of Neurosurgery, General Hospital Wiener Neustadt, Wiener Neustadt, Austria; 14 Department of Neurosurgery, University Hospital Ostrava, Ostrava, Czech Republic; 15 Department of Neurosurgery, Ceske Budejovice Hospital, Ceske Budejovice, Czech Republic; 16 Department of Neurosurgery, Pilsen University Hospital, Pilsen, Czech Republic; 17 Department of Neurosurgery, Motol University Hospital, Motol, Czech Republic; 18 Department of Neurosurgery, University Hospital Hradec Kralove, Hradec Kralove, Czech Republic; 19 Unit of Neurosurgery, Municipal Hospital - Ostrava Fifejdy, Ostrava Fifejdy, Czech Republic; 20 Department of Neurosurgery, University Hospital Kralovske Vinohrady, Prague, Czech Republic; 21 Department of Neurosurgery, University Hospital Olomouc, Olomouc, Czech Republic; 22 Department of Neurosurgery, Usti Nad Labem Hospital, Prague, Czech Republic; 23 Department of Neurosurgery, Liberec Hospital, Prague, Czech Republic; 24 Unit of Neurosurgery, Homolka Hospital, Prague, Czech Republic; 25 Department of Neurosurgery, University Hospital Brno and Masaryk University, Prague, Czech Republic; 26 Unit of Neurosurgery, Zlin Hospital, Prague, Czech Republic; 27 Unit of Neurosurgery, Pardubice Hospital, Prague, Czech Republic; 28 Department of Neurosurgery, St. Anne’s University Hospital Brno and Masaryk University, Prague, Czech Republic; 29 Department of Neurosurgery, Kantonspital St. Gallen, St. Gallen, Switzerland; 30 Department of Neurosurgery, Clinical Neuroscience Center, University Hospital Zurich, Zurich, Switzerland; 31 Clinic of Neurosurgery, Neurocenter of Southern Switzerland, Lugano, Switzerland; 32 Department of Neurosurgery, Kantonspital Aarau, Aarau, Switzerland; 33 Department of Neurosurgery, Geneva University Hospitals, Geneva, Switzerland

**Keywords:** Neuroscience, Health care, Medical research

## Abstract

The world currently faces the novel severe acute respiratory syndrome coronavirus 2 pandemic. Little is known about the effects of a pandemic on non-elective neurosurgical practices, which have continued under modified conditions to reduce the spread of COVID-19. This knowledge might be critical for the ongoing second coronavirus wave and potential restrictions on health care. We aimed to determine the incidence and 30-day mortality rate of various non-elective neurosurgical procedures during the COVID-19 pandemic. A retrospective, multi-centre observational cohort study among neurosurgical centres within Austria, the Czech Republic, and Switzerland was performed. Incidence of neurosurgical emergencies and related 30-day mortality rates were determined for a period reflecting the peak pandemic of the first wave in all participating countries (i.e. March 16th–April 15th, 2020), and compared to the same period in prior years (2017, 2018, and 2019). A total of 4,752 emergency neurosurgical cases were reviewed over a 4-year period. In 2020, during the COVID-19 pandemic, there was a general decline in the incidence of non-elective neurosurgical cases, which was driven by a reduced number of traumatic brain injuries, spine conditions, and chronic subdural hematomas. Thirty-day mortality did not significantly increase overall or for any of the conditions examined during the peak of the pandemic. The neurosurgical community in these three European countries observed a decrease in the incidence of some neurosurgical emergencies with 30-day mortality rates comparable to previous years (2017–2019). Lower incidence of neurosurgical cases is likely related to restrictions placed on mobility within countries, but may also involve delayed patient presentation.

## Introduction

The world currently faces a severe acute respiratory syndrome coronavirus 2 (SARS-CoV-2) pandemic, known as COVID-19. Following the initial outbreak, health care workflow was altered globally in many aspects^1,2^. Many countries placed elective surgical interventions on hold as a precaution to reduce its spread^3–5^. Non-elective, emergency surgeries, however, were continued as needed with revised management practices to protect patients, their families, and health care providers^6^. Introduced modifications included changes to work sequences in out- and inpatient services, pre- and perioperative changes, and faculty contingency planning^7^. As acute neurosurgical care involves both live-saving procedures and semi-urgent interventions to avoid lasting neurologic deficits, the triage of non-elective neurosurgical indications can pose a particular challenge under unprecedented circumstances, and potentially limited intensive care resources^8^.

In response to COVID-19, an abundance of viewpoints, guidelines, and reviews on best surgical practices during a pandemic have been published^3,9–16^. Comparatively fewer original research studies have documented the impact of the current pandemic on the incidence and surgical outcomes of non-elective cases. They have, to this point, focused on complications arising in patients with COVID-19 following surgery^17–23^. Notably lacking are studies exploring whether the systemic response to COVID-19 altered incidence and outcomes for uninfected patients undergoing non-elective/emergent surgical procedures. This information is critical to evaluate protocols for the ongoing second wave of COVID-19^24,25^.

The aim of the current study was to examine incidence and outcomes for non-elective emergent neurosurgical cases in COVID-19-negative patients during the first wave of the pandemic. A retrospective analysis was performed of most neurosurgical procedures across three European countries (Austria, the Czech Republic, and Switzerland) during the initial height of the COVID-19 pandemic (March 16 to April 15, 2020). The primary analysis addressed incidence and 30-day mortality in non-elective neurosurgical cases compared to three previous years.

## Methods

Our analysis included data from all neurosurgical centres in the Czech Republic, and the majority in Austria (10 of 11) and Switzerland (6 of 7), thus covering a population of almost 30 million people. During the outbreak these countries postponed elective surgeries, and intensive care resources were redistributed or reserved. Attempts were made to continue non-elective surgeries; however, in the absence of guidelines, it was unclear which procedures needed to be performed and what time frames were acceptable to initiate intervention. Despite the prevalence of COVID-19 cases in these countries, there were no obvious shortages in intensive care capacities during this time. Notably, the work flow was influenced heavily in the early phase of the pandemic with all related uncertainties. Information on all non-elective, emergent cranial and spinal neurosurgical procedures performed during March 16th–April 15th, 2020 was collected. This period was selected to reflect the culmination of the pandemic in these areas. Noteworthy, similar restrictions were applied in all assessed countries over the selected time period. Cases from identical time periods in 2017–2019 were analysed for reference. Ethical approval was obtained by the coordinating centres from each country (Ethikkommission Nummer der Medizischen Universität Innsbruck 1110/2020 in Austria, Etická komise FN Ostrava 448/2020 in the Czech Republic, and Kantonale Ethikkommission für die Forschung Bern 2020–01,433 in Switzerland). All research was performed in accordance with the local regulations as well as in Accordance with the declaration of Helsinki. Informed consent was waived by the approving committees.

### Patients

Demographic and surgical data were retrospectively collected in all participating centres. Demographic data elements included age, sex, and 30-day mortality. Conservatively managed patients were not included in our study. Inclusion/exclusion criteria are shown in Table 1. Cases were defined as: 1) traumatic brain injury (TBI) requiring any emergent neurosurgical intervention (i.e. monitoring for intracranial pressure, decompressive craniectomy, etc.), 2) chronic subdural hematomas (cSDH) in need of surgical evacuation due to the mass effect and/or neurological symptoms, 3) aneurysmal or non-aneurysmal subarachnoid haemorrhage, and other neurovascular pathologies, including arteriovenous malformations, arteriovenous fistula, and cavernous malformation requiring rapid treatment (i.e. latest within 2 weeks after onset of symptoms), 4) spinal procedures requiring non-elective surgical management involving degenerative spine disease with new onset or acute worsening of neurological symptoms (motor deficits and/or cauda equina syndrome, signs of spinal cord compression), infectious spine disease, spine tumours including metastasis, extradural, intradural and intramedullary tumours, as well as traumatic spine injuries with or without neurological symptoms, 5) new onset hydrocephalus or acute worsening in pre-existing hydrocephalus (e.g. shunt dysfunction), and 6) newly diagnosed intracranial lesions including low- or high-grade gliomas, meningioma, infarction in need for decompressive craniectomy, intracranial hematomas or abscesses requiring rapid or non-elective surgical scheduling (i.e. latest within 3 weeks after diagnosis or symptom onset).

**Table 1:** Inclusion and exclusion criteria.

Inclusion	Exclusion
Any traumatic brain injury requiring surgical intervention Chronic subdural hematoma requiring surgical evacuation (Aneurysmal and non-aneurysmal) Subarachnoid haemorrhage and other neurovascular pathologies (arteriovenous malformation, dural arteriovenous fistula, cavernoma) requiring endovascular and/or neurosurgical intervention Any (degenerative, traumatic, infectious, tumour) spine pathology requiring non-elective neurosurgical intervention New-onset or acute worsening in patients with hydrocephalus Intracranial lesions (including low- and high-grade gliomas, meningiomas, metastasis, infarction, intracranial hematoma, abscess) requiring non-elective surgical intervention	Conservative care Elective surgeries Any procedure not meeting predefined inclusion criteria

### Statistics

The number of total incident cases, and number within each condition, were determined in each year, separately for each country. Chi-square tests were used to test for differences in incidence across the years, and significant differences (p < 0.05) were further examined using standardized residuals. Rates of 30-day mortality were determined for each year, and within each condition separately for both countries. Thirty-day mortality was analysed using logistic regression with year (2020 vs. 2017–2019) as an independent variable. All models were adjusted for age (0–18, 18–39, 40–64, 65–74, and 75 + years old) and sex.

## Results

The participating centres in Austria, the Czech Republic, and Switzerland recorded 1,631, 2,234, and 887 non-elective neurosurgical cases, respectively. Female patients represented 44% of the sample in Austria and Switzerland, and 41% in the Czech Republic. The median age in Austria and the Czech Republic was 61 years (range: 0–95 in Austria, 0–96 in the Czech Republic), and 65 for Switzerland (17–96). One Austrian centre could only provide information on spine cases for 2019 and 2020, and thus was removed from descriptions and analyses of total cases and spine cases, but was included for the other conditions.

### Incidence

The overall incidence of neurosurgical cases trended downwards in Austria and the Czech Republic in 2020. Chi-squared analyses revealed that significant differences in incidence rates occurred for cSDH, spine, acute hydrocephalus, and tumour cases in Austria, and in TBI and cSDH in the Czech Republic. These differences were driven by lower incidences in 2020 for all conditions in Austria, excluding tumours and other intracranial lesions. In the Czech Republic, TBI increased in 2019, then reached a 4 year low in 2020. There were no significant trends for incident cases in Switzerland (Table 2).

**Table 2:** The incidence of non-elective neurosurgical cases in Austria, Czech Republic and Switzerland from March 16th until April 15th 2017–2020.

	2017	2018	2019	2020	*P*-value*
**Austria**					
Total cases	400	417	392	345	0.06
**Condition**					
TBI	42	43	26	27	0.06
cSDH	72	63	62	38	0.01
SAH	26	47	37	41	0.10
Spine	104	110	111	71	0.01
Acute hydrocephalus	59	42	58	28	0.003
Tumour and other intracranial lesions	105	126	105	152	0.007
**Czech Republic**					
Total cases	586	551	584	513	0.10
**Condition**					
TBI	74	68	98	48	< 0.001
cSDH	87	110	84	53	0.002
SAH	59	59	66	63	0.90
Spine	175	139	135	153	0.09
Acute hydrocephalus	53	42	59	45	0.31
Tumour and other intracranial lesions	138	133	142	151	0.74
**Switzerland**					
Total cases	243	220	208	216	0.38
**Condition**					
TBI	22	24	16	18	0.57
cSDH	38	37	36	41	0.95
SAH	30	14	21	18	0.08
Spine	61	64	47	49	0.27
Acute hydrocephalus	24	27	24	26	0.97
Tumour and other intracranial lesions	68	54	64	64	0.63

*TBI* Traumatic brain injury, *cSDH* chronic subdural hematoma, *SAH* subarachnoid haemorrhage and other vascular pathologies.

*P-values derived from chi-square tests.

### 30-day mortality

Of the recorded cases in Austria and the Czech Republic, 1,616 (99% of total) in Austria, 2,222 (99%) in the Czech Republic, and 875 (97%) in Switzerland, were included in the logical regression analysis of 30-day mortality. Logistic regression models revealed that the year 2020 was not significantly associated with any increased odds of 30-day mortality. In fact, TBI within the Czech Republic had a significant lowered 30-day mortality (Tables 3 and 4).

**Table 3:** The 30-day mortality rates from non-elective neurosurgical cases in Austria, Czech Republic and Switzerland from 2017–2020.

	2017	2018	2019	2020
**Austria**				
All conditions	6.0%	6.0%	3.6%	6.5%
**Condition**				
TBI	12.2%	14.3%	3.8%	7.7%
cSDH	2.9%	1.6%	0.0%	5.3%
SAH	8.0%	13.3%	16.2%	9.8%
Spine	0.0%	0.9%	0.0%	1.4%
Acute hydrocephalus	8.5%	0.0%	3.4%	7.4%
Tumour and other intracranial lesions	9.7%	8.7%	6.7%	8.7%
**Czech Republic**				
All conditions	5.1%	7.1%	8.4%	4.5%
**Condition**				
TBI	13.5%	20.9%	17.3%	4.3%
cSDH	2.3%	4.5%	6.0%	5.9%
SAH	15.3%	5.1%	6.1%	10.0%
Spine	1.1%	2.9%	3.7%	1.3%
Acute hydrocephalus	5.7%	9.5%	8.5%	6.7%
Tumour and other intracranial lesions	2.9%	6.8%	9.3%	4.6%
**Switzerland**				
All conditions	7.7%	6.9%	9.8%	8.9%
**Condition**				
TBI	18.2%	13.0%	18.8%	16.7%
cSDH	0.0%	0.0%	2.9%	2.4%
SAH	24.1%	21.4%	23.8%	16.7%
Spine	1.8%	1.6%	4.3%	6.3%
Acute hydrocephalus	4.2%	11.1%	16.7%	16.0%
Tumour and other intracranial lesions	7.5%	9.3%	7.8%	7.8%

*TBI* Traumatic brain injury, *cSDH* chronic subdural hematoma, *SAH* subarachnoid haemorrhage and other vascular pathologies.

**Table 4:** Logistical regression of 30-day mortality in non-elective neurosurgical cases in Austria, Czech Republic and Switzerland. Effects estimates reflection 2020 compared to aggregate of reference years (2017–2019).

Country	Condition	Effect (95% CI)	P-value*
Austria	All	1.25 (0.74–2.03)	0.38
	TBI	0.67 (0.10–2.77)	0.62
	cSDH	4.06 (0.50–27.79)	0.15
	SAH	0.72 (0.19–2.17)	0.58
	Spine	4.94 (0.19–128.30)	0.26
	Acute hydrocephalus	2.19 (0.30–10.39)	0.36
	Tumour and other intracranial lesions	1.02 (0.49–2.02)	0.95
Czech Republic	All	0.65 (0.40–1.02)	0.07
	TBI	0.20 (0.03–0.70)	0.03
	cSDH	1.25 (0.27–4.20)	0.74
	SAH	1.17 (0.40–3.02)	0.75
	Spine	0.52 (0.08–1.97)	0.39
	Acute hydrocephalus	0.74 (0.16–2.54)	0.66
	Tumour and other intracranial lesions	0.73 (0.28–1.67)	0.49
Switzerland	All	1.12 (0.63–1.91)	0.68
	TBI	1.80 (0.34–8.15)	0.45
	cSDH	3.46 (0.13–91.7)	0.39
	SAH	0.39 (0.07–1.67)	0.24
	Spine	5.08 (0.88–28.55)	0.06
	Acute hydrocephalus	1.53 (0.37–5.59)	0.53
	Tumour and other intracranial lesions	1.00 (0.31–2.77)	1.00

*CI* confidence interval, *TBI* Traumatic brain injury, *cSDH* chronic subdural hematoma, *SAH* subarachnoid haemorrhage and other vascular pathologies.

*All models adjusted for age and sex.

## Discussion

Our observations indicate a general trend toward reduced numbers of non-elective, emergency neurosurgical cases during the COVID-19 pandemic in Austria, Switzerland and the Czech Republic. This was primarily driven by a decreased incidence of conditions commonly associated with traumatic aetiologies, including brain injuries, spine conditions, and chronic subdural hematomas. Although variable across the study period (2017–2020), 30-day mortality during COVID-19 in Austria, the Czech Republic, and Switzerland did not significantly increase overall or for any condition compared to recent prior years. Despite the challenges of delivering health services and intensive care during the COVID-19 pandemic^8,26^, our data indicate that emergency neurosurgical care could still be provided in these three countries.

The decreased incidence of selected neurosurgical conditions does not come as a surprise during a time when Austria the Czech Republic and Switzerland imposed relatively similar societal “lock down” measures to reduce the spread of COVID-19. Nevertheless, based on data representative of three European countries, this study is the first to quantify the extent to which COVID-19 impacted emergent surgical practice. The most obvious explanation is restricted mobility and outdoor activity within countries. This, in turn, led to fewer traumatic accidents (e.g., motor vehicle) and a subsequent reduction in brain and spinal cord injuries^27–29^.

On the other hand, the lower incidence of acute hydrocephalus and spine cases in Austria plus the reduced number of interventions for cSDH warrants further attention. Chronic SDH is thought to result from minor trauma weeks or months earlier in the elderly and may thus not reflect traumatic injuries in the lock-down period but rather delayed presentation of patients to health care providers^28^. This may be particularly applicable for an insidious disease like cSDH. Thus, there is the possibility that individuals with signs of symptoms of these conditions were hesitant to seek medical care during COVID-19. This was reported as a potential factor in Northern Italy in patients with acute coronary syndrome^30^. Faced with concerns and uncertainty regarding transmission of COVID-19, staying home may reasonably have been perceived as a safer alternative to treatment for symptoms such as headaches, dizziness, and confusion related to trauma. While this is a difficult hypothesis to explicitly test with our data, the incidence of non-traumatic cases did not significantly decrease in 2020 compared to reference years in either Austria, the Czech Republic or Switzerland (2017–2019). This is particularly notable for the incidence of tumours, which paradoxically increased in Austria in 2020. Had a reduction in the incidence of tumours been observed, one might reasonably speculate that reluctance to seek medical care served as a contributing factor; in the absence of such an effect, reduced incidence is most likely attributable to mobility restrictions imposed at the societal level that led to fewer traumatic injuries requiring neurosurgical intervention^27^. Importantly, all analysed countries were not as severely hit by the pandemic as others, and intensive care capacities were available across all regions. Further, most non-elective cases need intensive care measures postoperatively. Neurological manifestations of the COVID-19 pandemic need to be kept in mind as well^31,32^.

From a health care delivery standpoint, it was very encouraging to find that the odds of 30-day mortality did not increase in 2020 in these countries, for any condition. Due to altered management practices, issues related to the quality of care for patients with neurooncological diseases during the pandemic have been raised^33^. Our results, however, suggest that methods imposed to deliver care during COVID-19 in Austria, the Czech Republic, and Switzerland did not have major negative consequences.

Major strengths of this study were the large sample size, inclusion of multiple reference periods (2017–2019) for comparison to COVID-19, and representation of nearly all neurosurgical centres in Austria, the Czech Republic, and Switzerland. The interpretation of our data is limited, however, by the observational and retrospective nature of the study. Further, only patients that received surgical care were analysed. Future studies should consider the extent to which societal measures associated with COVID-19 may have influenced access to health care services by exploring other outcomes, including disease severity. Additionally, lengthening the duration of data acquisition beyond the 1-month window would allow the detection of a “rebound” in cases that were reduced by the pandemic. Hence, surgical prioritization after the pandemic is important too^34^. Further, one should keep in mind that COVID-19 per se causes significant neurological manifestations^35,36^ and some patients might need surgical care.

## Conclusion

The world continues to face major challenges in managing COVID-19, which also include potential restrictions of health care services. Hence, it is uplifting to know that the neurosurgical community’s response to the initial phase of the pandemic, in at least three European countries, maintained high standards of care and low rates of acute mortality. Nevertheless, the potential of delayed patient presentation warrants further investigation as emergent neurosurgical care needs to be provided at all times.
